# Single-Nucleotide Polymorphisms Sequencing Identifies Candidate Functional Variants at Prostate Cancer Risk Loci

**DOI:** 10.3390/genes10070547

**Published:** 2019-07-18

**Authors:** Peng Zhang, Lori S. Tillmans, Stephen N. Thibodeau, Liang Wang

**Affiliations:** 1 Department of Pathology, MCW Cancer Center, Medical College of Wisconsin, 8701 Watertown Plank Road, Milwaukee, WI 53226, USA; 2 Department of Laboratory Medicine and Pathology, Mayo Clinic College of Medicine, 200 First Street SW, Rochester, MN 55905, USA

**Keywords:** SNPs-seq, eQTL, prostate cancer, SNP, EMSA

## Abstract

Genome-wide association studies have identified over 150 risk loci that increase prostate cancer risk. However, few causal variants and their regulatory mechanisms have been characterized. In this study, we utilized our previously developed single-nucleotide polymorphisms sequencing (SNPs-seq) technology to test allele-dependent protein binding at 903 SNP sites covering 28 genomic regions. All selected SNPs have shown significant cis-association with at least one nearby gene. After preparing nuclear extract using LNCaP cell line, we first mixed the extract with dsDNA oligo pool for protein–DNA binding incubation. We then performed sequencing analysis on protein-bound oligos. SNPs-seq analysis showed protein-binding differences (>1.5-fold) between reference and variant alleles in 380 (42%) of 903 SNPs with androgen treatment and 403 (45%) of 903 SNPs without treatment. From these significant SNPs, we performed a database search and further narrowed down to 74 promising SNPs. To validate this initial finding, we performed electrophoretic mobility shift assay in two SNPs (rs12246440 and rs7077275) at *CTBP2* locus and one SNP (rs113082846) at *NCOA4* locus. This analysis showed that all three SNPs demonstrated allele-dependent protein-binding differences that were consistent with the SNPs-seq. Finally, clinical association analysis of the two candidate genes showed that *CTBP2* was upregulated, while *NCOA4* was downregulated in prostate cancer (*p* < 0.02). Lower expression of *CTBP2* was associated with poor recurrence-free survival in prostate cancer. Utilizing our experimental data along with bioinformatic tools provides a strategy for identifying candidate functional elements at prostate cancer susceptibility loci to help guide subsequent laboratory studies.

## 1. Introduction

Prostate cancer is the second most common cancer and the fifth leading cause of cancer death among men, with almost 1.3 million new cases and 359,000 associated deaths in 2018 worldwide [1]. Histological phenotypes of prostate cancer include adenocarcinoma, squamous cell carcinoma, and neuroendocrine carcinoma. Risk factors of prostate cancer involve age, genetics (family history and ethnicity), environmental and lifestyle (smoking and alcohol consumption), and gene–environment interaction [2,3,4]. 

To identify genetic determinants of prostate cancer risk, over 39 genome-wide association studies (GWAS) on prostate cancer have reported approximately 482 unique prostate cancer risk single-nucleotide polymorphisms (SNPs), based on the NHGRI-EBI catalogue of published GWASs (http://www.ebi.ac.uk/gwas) [5,6]. Importantly, a significant number of these reported SNPs have subsequently been validated in well-powered case–control studies. However, potential causal variants and their biological mechanisms at risk loci are largely unknown, even though many post-GWAS studies have unraveled new gene networks and signaling pathways associated with germline variants [7]. Because these risk SNPs have been found in noncoding regions of the genome, it is believed that many of these risk SNPs are located in regulatory domains of the genome that control gene expression rather than in coding regions that directly affect protein function [5,6,8]. 

To molecularly characterize these risk loci, causal SNPs and their target genes have to be identified [6,7]. Expression quantitative trait loci (eQTL) has been widely used to identify SNP–gene association [9]. Previously, we have established a 471 normal prostate tissue-specific eQTL dataset and examined 100 prostate cancer risk regions. We have identified 51 regions with significant eQTL signal that involved 88 genes [10]. Due to a large number of SNPs in each risk loci, however, high-throughput screening tools are needed to identify causal SNPs. Recently, we developed a high-throughput single-nucleotide polymorphisms sequencing (SNPs-seq) technology that takes advantage of the higher retention rate of protein-bound DNA oligos in protein purification system to quantitatively sequence SNP-containing oligos. We have applied the SNPs-seq to simultaneously screen 374 risk SNPs for their allele-dependent protein-binding differences [11]. In the new study, we performed more comprehensive SNPs-seq analysis that covered 903 SNPs at 28 prostate cancer risk loci. 

## 2. Materials and Methods 

### 2.1. Selection of Candidate SNPs

**Figure 1:**
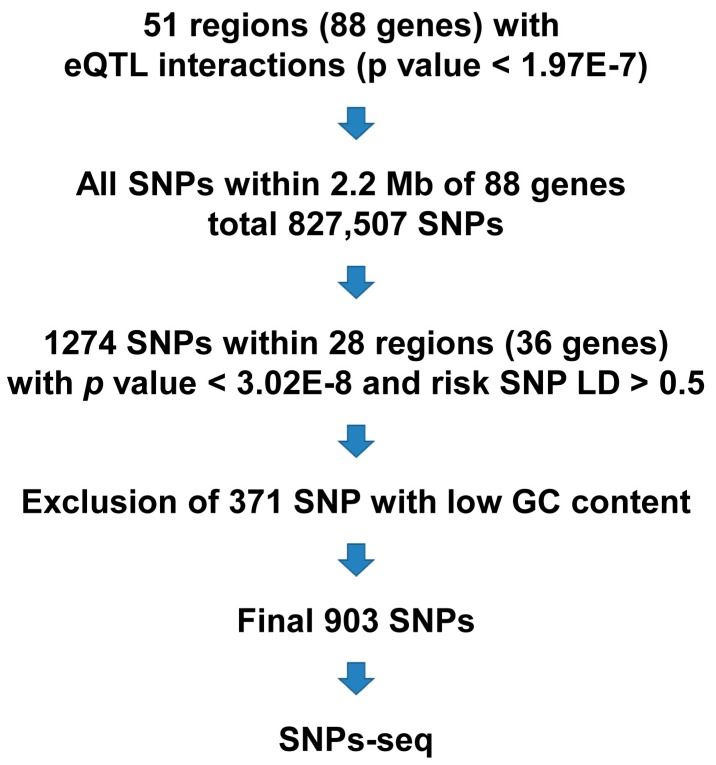
Workflow of candidate single-nucleotide polymorphism (SNP) selection. eQTL: expression quantitative trait loci.

In our previous study [10], we identified 51 regions that showed significant eQTL signals in 88 genes. From the 51 regions with eQTL *p* value < 1.97 × 10^−7^, we extracted all SNPs within 2.2 Mb of these 88 genes and identified a total of 827,507 SNPs. We narrowed this down to 1274 SNPs from 28 regions (36 genes) with risk SNP LD > 0.5 and *p* value < 3.02 × 10^−8^ for further study. Based on our previous publication [11], we further excluded SNP sites with low GC content and finalized 903 SNPs for SNPs-seq analysis (Figure 1 shows workflow of candidate SNP selection, Table 1 shows distribution of SNPs in 28 regions, and Table S1 shows the list of 903 SNPs). 

### 2.2. Cell Culture and Nuclear Extraction

We obtained the human prostate cancer cell line LNCaP from the American Type Culture Collection (Manassas, VA, USA). Cells were cultured in a 10 cm dish with RPMI-1640 containing 10% fetal bovine serum (FBS) and 1% penicillin/streptomycin (Thermo Fisher Scientific, Waltham, MA, USA). For androgen treatment, the medium was replaced by phenol red-free RPMI supplemented with 10% charcoal-stripped FBS (CS-FBS) to achieve deprivation of steroid hormones and growth factors. Twenty-four hours after hormone depletion, the cells were treated with 10 nM dihydrotestosterone (DHT) or 0.1% ethanol (ETH) for 48 hours. We extracted the nuclear protein using Ne-Per nuclear and cytoplasmic extraction reagents (Thermo Fisher Scientific). Protein concentrations were determined using Pierce BCA (bicinchoninic acid) protein assay kit (Thermo Fisher Scientific). The nuclear protein extracts were aliquoted at 25 μL each and stored at −80 °C until use. 

### 2.3. SNPs-seq 

For each selected candidate SNP, we synthesized 4 single-strand oligos (variant and reference allele, +/− strand) at 20 μM (21 bp/oligo, SNP in the middle) in 25 μL duplex buffer (Integrated DNA Technologies, Coralville, IA, USA). For oligo annealing, we mixed 10 μL of +/− strand oligos to one well and performed initial denaturation at 95 °C for 3 min, followed by gradual temperature decrease from 95 to 25 °C in 70 min. We pooled all ds-oligos by pipetting 2 μL of each ds-oligo. 

For oligo–protein binding assay, we mixed 5 μL ds-oligos pool (~400 ng), 5 μL nuclear extract (~10 μg), and 15 μL binding buffer. After 30 min of incubation at room temperature (20–23 °C), the binding reaction mixtures were passed through the isolation column (Signosis, Santa Clara, CA, USA). After washing 4 times, the protein-bound oligos were collected in 60 μL elution buffer. The collected oligos were further purified using Oligo Clean kit (Zymo research, Irvine, CA, USA). Concentration of the purified oligos was determined using Qubit dsDNA HS Assay kit (Thermo Fisher Scientific). Two repeats were used in each experimental condition to ensure reproducibility and to minimize technical variability. 

We prepared sequencing libraries using ThruPLEX DNA-seq kit (Takara Bio, Mountain View, CA, USA) with 2 ng purified eluted oligos. After 15-cycle amplification, the libraries were purified using SPRIselect reagent (Beckman Coulter Life Sciences, Indianapolis, IN, USA) and quantified by Qubit. The libraries were sequenced on Illumina HiSeq2500 with 50 bp single read. A total of 903 SNP sites with 1806 unique sequences were used as template. DNASTAR Genomic Suite was used for sequence mapping and read counting. Only perfect match was allowed during mapping. To determine the allelic protein-binding difference, we calculated the biased allelic binding (BAB) score using this formula: BAB = log2 [test(RC_variant_/RC_reference_)/input(RC_variant_/RC_reference_)]. The test and input represented tested (DHT/ETH) and input control samples, respectively. RC_variant_/RC_reference_ represents the ratio of read counts from variant allele and reference allele. The sequences data of SNPs-seq are available upon request. 

### 2.4. Database Search for Potential Functional SNPs

We took advantage of several existing databases to identify potential functional SNPs. First, we searched Regulome DB [12] to identify DNA features and regulatory elements that intersected the coordinate of the SNPs. Then, we used HaploReg [13] to explore chromatin states, conservation, and regulatory motif alterations within sets of genetically linked variants. We also searched published ChIP-seq (chromatin immunoprecipitation sequencing) data for transcription factor (TF) binding at the SNPs [14,15]. Furthermore, we used SNPnexus [16] to screen ENCODE [17] and other datasets for functional annotation of the candidate SNPs. Moreover, we applied the Variant Effect Predictor (VEP) toolset to determine the effect of candidate SNPs on genes, transcripts, and protein sequence, as well as regulatory regions [18]. Lastly, we used MATCH [19] tool to find putative TF binding sites at the SNP sequences.

### 2.5. Electrophoretic Mobility Shift Assay (EMSA) 

To validate the allelic protein-binding difference, we performed electrophoretic mobility shift assay (EMSA) using the LightShift Chemiluminescent EMSA kit (Thermo Fisher Scientific). All oligonucleotides were synthesized from Integrated DNA Technologies. Target oligonucleotide (length 29 bp, SNPs in the middle) was 3’ end labeled with biotin using Biotin 3’ End DNA Labeling kit (Thermo Fisher Scientific). The binding reaction mixtures (20 μL) included 1X binding buffer, 1 μL poly (dI-dC), 3 μL of nuclear extract, 100 fmol of 3’ end-labeled oligo, and 200-fold excess of unlabeled oligo for the competitive assay. Reaction mixtures were subjected to electrophoresis using 6% polyacrylamide gel (0.5X TBE) and then transferred onto Biodyne B Nylon Membrane (Thermo Fisher Scientific). Protein–DNA binding was detected using the Chemiluminescent Nucleic Acid Detection Module. The blot was visualized by C-DiGit Blot Scanner and analyzed by Image Studio Software (LI-COR Biotechnology, Lincoln, NE, USA). 

### 2.6. Clinical Association Analysis

To evaluate the clinical association of selected genes, we analyzed TCGA prostate cancer dataset [20]. We used the Mann–Whitney test to compare gene expression between normal and prostate cancer tissues. We applied log-rank test to assess the association between recurrence-free survival (RFS) and gene expression. Samples were stratified into two groups based on the mean values of gene expression levels. GraphPad was used to perform statistical analyses. 

## 3. Results

### 3.1. High-Quality SNPs-seq Libraries

**Figure 2:**
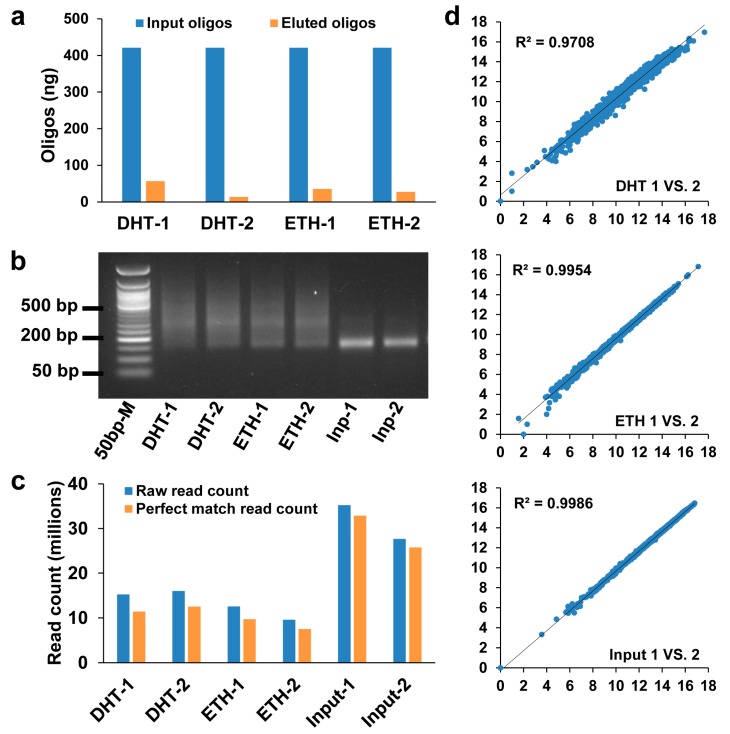
Quality control of SNPs-seq. (**a**) Eluted oligos after protein–DNA binding assay accounted for 3–13% of original input oligos. (**b**) Sizes of SNPs-seq libraries were ~160 bp in the input group and 150–500 bp in the test group. (**c**) Raw read counts and perfect match read counts in test and input samples. (**d**) Correlation of raw read count at log2 values between technical replicates in dihydrotestosterone (DHT), ethanol (ETH), and input samples.

To perform protein–DNA binding assay, we used ~400 ng ds-oligos pool as input. After incubation and extensive washing, we purified the protein-bound oligos. Quantification analysis showed that 3–13% (10–50 ng) of original input was protein-bound (Figure 2a). Because the ds-oligos were 21 bp and adaptors were 140 bp, the length of libraries were estimated to be ~160 bp. As expected, the input libraries had a clear sharp band at ~160 bp (Figure 2b). However, sizes of DHT/ETH libraries distributed in a relatively wide range from 150 to 500 bp. This could be explained by nuclear extracts that contain fragmented DNAs from cell nuclei. To estimate mappable rate, we counted sequence reads with a perfect match to one of 1806 oligo template sequences and found that the mappable rate was 75–78% for test samples and 93% for input controls (Figure 2c). We also tested reproducibility for each sample and observed high correlation between two technical replicates (R^2^ > 0.97) (Figure 2d). To test the reproducibility between different studies, we compared read counts of 116 SNP sequences that were shared between this study and previous SNPs-seq [11] and observed significant correlation (R^2^ > 0.73) (Figure S1). 

### 3.2. Candidate SNPs with Allele-Dependent Protein Binding

**Figure 3:**
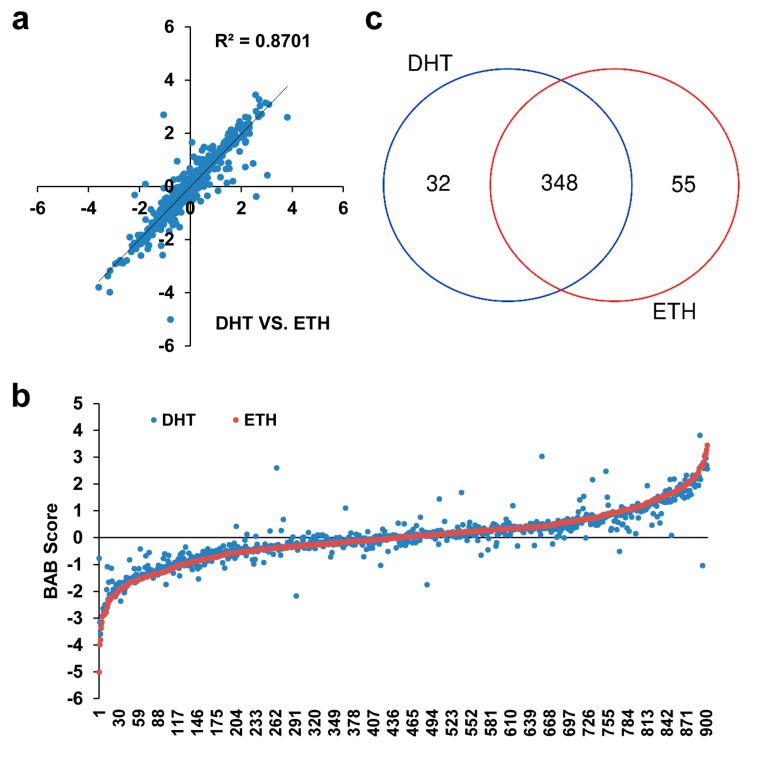
Correlation and distribution of biased allelic binding (BAB) score. (**a**) Correlation of BAB scores between DHT and ETH groups. (**b**) BAB score distribution in DHT and ETH groups (sorted by BAB score from low to high in ETH group). (**c**) Overlap of significant SNPs (absolute BAB score ≥ 0.58) between DHT and ETH groups.

To determine the allele-specific protein binding, we calculated the BAB score using read count ratio between variant allele and reference allele. The BAB score showed a wide-range distribution from −5.02 to 3.81 and demonstrated a strong correlation between DHT- and ETH-treated samples (R^2^ > 0.87) (Figure 3a). When sorting BAB score from low to high in ETH samples, the BAB score in DHT samples clearly tended to the same direction (Figure 3b). When defining absolute BAB score ≥ 0.58 (meaning 1.5-fold difference) as significant cutoff, 380 (42%) and 403 (45%) SNPs showed significant difference in DHT-treated samples and in ETH-treated samples, respectively. Among those, 348 (39%) SNPs were shared between two treatment conditions, 32 SNPs were specific under DHT treatment, and 55 SNPs were specific under ETH treatment (Figure 3c, Tables S1 and S2). Interestingly, some of these SNPs have been published as functional candidates in previous studies, including rs13215402 (RGS17) [11], rs6579003 and rs7123299 (ASCL2) [11], rs10993994 (MSMB) [21], and rs4907792 (NUDT11) [14]. 

To estimate whether the 348 shared SNPs were resided in known enhancer regions, we performed association tests using online tools including GREAT [22] and EnhancerAtlas [23]. The GREAT assigns biological meaning to a set of noncoding genomic regions by analyzing the annotations of the nearby genes. EnhancerAtlas provides a set of useful analytic tools that allow users to query and compare enhancers in a particular genomic region or associated with a gene of interest and assign enhancers and their target genes from a custom dataset. When GREAT was applied to annotate the 348 SNPs with nearby genes, we observed that the absolute distance to the transcription start site (TSS) from most SNPs were 5–50 kb (43.45%) and 50–500 kb (44.36%) and more likely resided in enhancer regions (Figure S2). When EnhancerAtlas tool was applied, we found 128 (36.78%) SNP sites at known enhancer regions and 83 (23.85%) SNP sites at known promoter regions in 179 human cell lines. For prostate cancer cell lines (LNCaP and VCaP), we found seven (2.01%) SNP sites at enhancer regions and 83 (23.85%) SNP sites at promoter regions. 

### 3.3. Candidate SNPs to Prioritize Functional Validation

**Figure 4:**
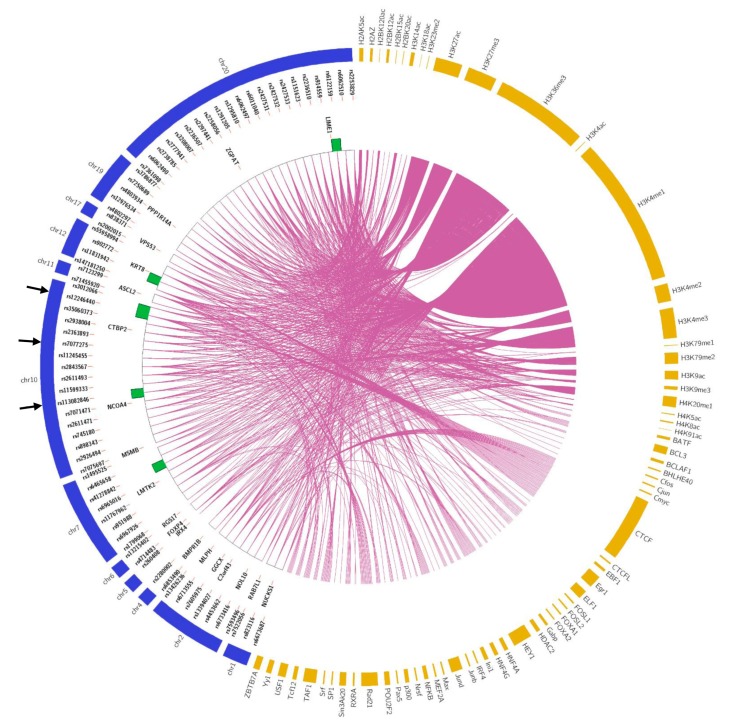
Circos plot showing functional annotations on the 74 promising SNPs. Left semicircle: from outer to inner ring are chromosomes and related SNPs, genes, and MATCH results (putative transcription factor binding sites); right semicircle: histone and transcription factor marks from Regulome DB, HaploReg, SNPnexus, and Variant Effect Predictor (VEP). Arrows (→) indicate three selected SNPs for electrophoretic mobility shift assay (EMSA) validation.

To facilitate discovery of functional candidates on the 348 significant SNPs in both DHT and ETH treatment conditions, we applied Regulome DB and found 95 SNPs with Regulome DB score higher or equal to 4 (Table S3). Then, we searched multiple databases, including HaploReg, SNPnexus, and Variant Effect Predictor, for functional annotations at 348 SNP sites. These analyses revealed, among the 95 SNPs from Regulome DB, 74 promising functional SNPs with modifier impact, motifs change, and histone/transcription factor marks (Figure 4 and Figure S3, Table S3). Lastly, we applied MATCH program to identify potential TF binding proteins at selected SNP sequences. This analysis showed potential disruption of TF binding at six of the 74 SNP sites. For example, the SNP rs113082846 showed a strong eQTL signal with gene *NCOA4* (*p* value = 5.32 × 10^−52^). In the SNPs-seq analysis, the BAB score of alternate allele C to reference G allele was 2.95 in the DHT-treated sample and 3.14 in the ETH group. When the experimental oligo sequence with the C allele was entered in MATCH, a binding site for ELK1 (transcription activator) was reported with a core match of 1.000 and a matrix match of 0.988. No binding sites were reported for the G allele. These functional annotations on the 348 SNPs are listed in Table S3 and visualized in Figure 4 and Figure S3. 

### 3.4. Validation of Selected Candidate SNPs

**Figure 5:**
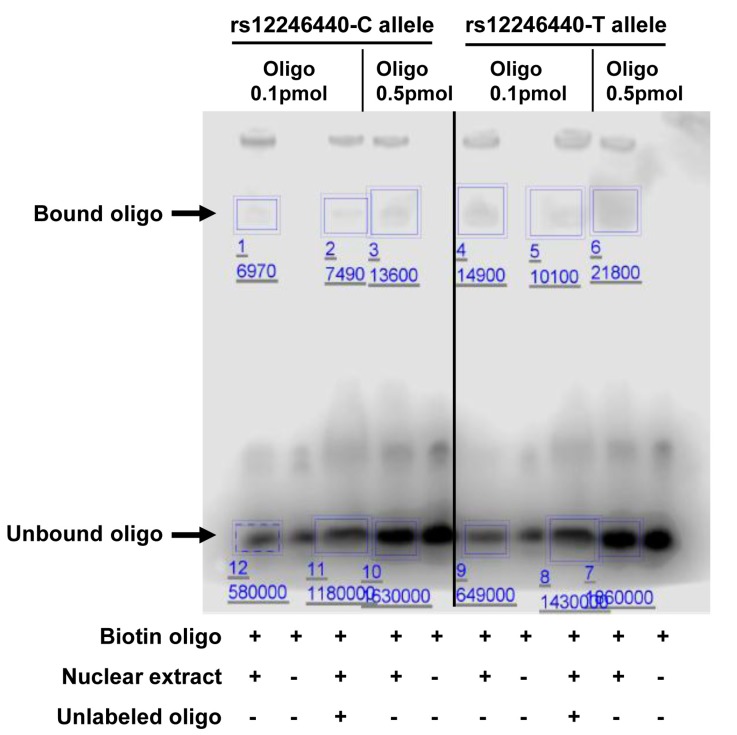
Validation of SNP rs12246440 by EMSA. Compared to T allele, C allele shows weaker protein binding in both 0.1 pmol and 0.5 pmol oligo. The binding signal is reduced by the addition of unlabeled oligos. The ratio of C to T allele from EMSA is consistent with ratio from SNPs-seq.

In principle, both EMSA and SNPs-seq utilize the same protein–DNA binding assay. To validate SNPs-seq results, we therefore applied EMSA to test allele-dependent protein binding in three SNP sites based on the BAB score and epigenomic annotations from other databases. The EMSA showed that SNP rs12246440 (chr10:125048307, intron variant of C-terminal binding protein 2, or *CTBP2*) had protein-binding difference between C and T allele. The C allele had lower binding ability than T allele. When unlabeled oligo was used, the signal was significantly reduced, suggesting specific oligo binding. This result was consistent with SNPs-seq (Figure 5 and Table 2). The EMSA also confirmed allele-specific binding at SNP rs7077275 (chr10:125008641, intron variant of *CTBP2*) and rs113082846 (chr10:46093020, intergenic variant, near nuclear receptor coactivator 4, or *NCOA4*) (Figures S4 and S5). The functional annotations of the three SNPs are shown in Table S4. 

### 3.5. Association of Candidate Genes with Clinical Outcomes

**Figure 6:**
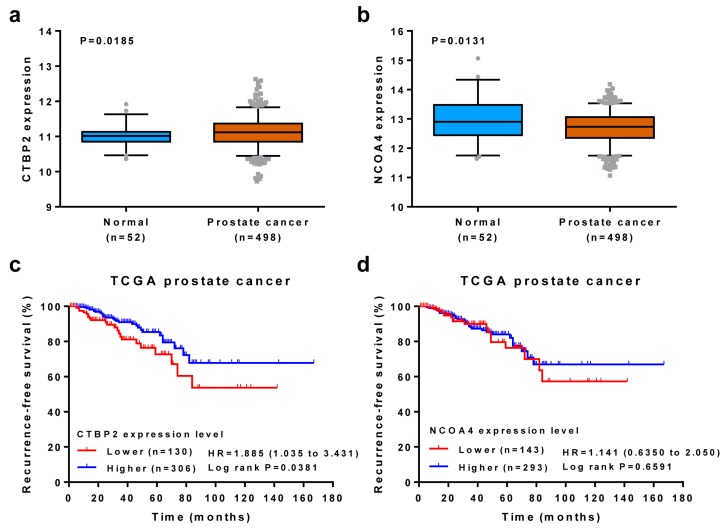
Clinical association of *CTBP2* and *NCOA4* in TCGA dataset. (**a,b**) Gene expression difference between normal and prostate cancer were determined using Mann–Whitney test. The upper, middle, and lower bounds of boxes represent the 75th, 50th, and 25th percentile of the values, respectively. The whiskers represent 95th to 5th percentile. (**c,d**) Association of recurrence-free survival (RFS) with gene expression was evaluated using log-rank test.

To associate candidate genes (*CTBP2* and *NCOA4*) with clinical outcomes, we examined gene expression and its association with survival using TCGA dataset. When compared to normal prostate tissues (N = 52), the *CTBP2* expression level was significantly higher in prostate cancer tissues (N = 498) (*p* = 0.0185) (Figure 6a), while the *NCOA4* expression was significantly lower in prostate cancer tissues (*p* = 0.0131) (Figure 6b). The log-rank test showed that lower *CTBP2* expression level was associated with poor RFS (*p* = 0.0381) (Figure 6c). However, we did not observe any survival association with *NCOA4* expression (*p* = 0.6591) (Figure 6d). To see if the two genes had any synergistic effect on survival, we combined expression values of *CTBP2* and *NCOA4*, weighted by their estimated regression coefficients, and performed the log-rank test. This analysis also showed the lower expression level associated with poor RFS (*p* = 0.0247) (Figure S6).

## 4. Discussion

GWASs have identified thousands of SNP associations with complex diseases and traits. In the post-GWAS era, however, there is still a significant challenge to functionally characterize these risk SNPs and their underlying biology [24]. In this study, we applied the SNPs-seq technology [11] to test 903 SNPs from prostate cancer risk SNPs-related eQTL data [10]. We reported a significant fraction of SNPs showing allelic difference for protein binding. Because prostate cancer is driven by a gain of function in AR (androgen receptor) that is usually accompanied by DHT to drive expression of AR-induced genes [25,26], we classified significant SNPs into three groups (DHT-specific, ETH-specific, and shared between the two) based on the BAB score and treatment conditions. We also performed a database search and further narrowed down the promising functional SNPs. In addition, we applied EMSA and validated a selected group of candidate SNPs. Finally, we associated the candidate genes with tissue type and recurrence-free survival. This study provides a list of candidate functional SNPs and will significantly enrich resources for functional annotation of prostate cancer risk SNPs.

To determine whether a SNP is functional, a common approach is to map the SNP to a regulatory element defined by ENCODE [17] and the Roadmap Epigenomics Program [27]. Although useful for mapping regulatory genomic regions, none of the datasets provide direct access to allelic binding preferences. To test whether candidate SNP are functional, EMSA [28] may be used to identify allele-specific protein binding. However, this low-throughput assay is not suitable when screening because hundreds to thousands of candidate SNPs are needed. ChIP-seq [29] is another method to identify allele binding difference at TF binding sites. However, this method requires heterozygous status at every tested SNP site, which is not possible for a specific cell line. To address this issue, we developed the SNPs-seq [11]. Our study shows that SNPs-seq is a powerful tool to identify allelic protein-binding difference with high-throughput capacity. Because GC content significantly affects read counts in the SNPs-seq assay [11], in this study, we excluded 371 SNPs with low GC content before the final oligo pooling. Additionally, it is worth mentioning that SNPs-seq is a screening tool, which does not recognize proteins that bind to the SNP-containing oligos. Further laboratory tests are needed to validate the findings. Moreover, the main goal of this study is not to identify a specific SNP that regulates prostate cancer-related gene. Instead, the study provides a key set of SNPs that can be prioritized for further functional studies. From the preselected promising SNP, several functional experiments, such as RNAi, CRISPR, and reporter assay, may be used for functional validation. In addition, the approaches used in this study are not limited to prostate cancer. They may be applied to characterize functional SNPs at any GWAS loci of any disease phenotype. 

In this study, we also provided candidate SNPs and their target genes. Among these are *CTBP2* and *NCOA4*. *CTBP2* is a binding partner for the E1A-transforming proteins. *CTBP2* exert transcriptional repression primarily via recruitment of a corepressor complex to DNA that consists of histone deacetylases and histone methyltransferases [30]. A joint GWAS showed that *CTBP2* for SNP rs4962416 was significantly associated with prostate cancer [31]. *CTBP2* is overexpressed in prostate cancer, and its increased expression is significantly correlated with malignant behaviors [32]. Silencing of *CTBP2* markedly increases apoptosis of prostate cancer cells; decreases the expression of *IL-8*, *AT2R*, *CCND1*, *MMP9*, *MYC*, and *HSPC111*; and reduces tumor growth in mouse xenograft model of human prostate cancer [32,33,34,35,36]. However, our analysis of TCGA data showed that high expression of *CTBP2* was associated with longer RFS in prostate cancer patients. Takayama et al. found that high *CTBP2* expression levels were correlated with poor cancer-specific survival in patients [35]. The reasons for the conflicting observation may involve different methods to determine the expression level, different patient population, and/or different clinical end points for survival analysis. *NCOA4*, also known as androgen receptor-associated protein 70 (*ARA70*), is a coactivator for a variety of nuclear receptors [37]. SNP rs10993994 (near *NCOA4*) is associated with prostate cancer, and the risk allele is associated with increased expression of five *NCOA4* isoforms in histologically normal prostate tissue [31,38]. The expression of *NCOA4* in human prostate tissues is not consistent. Some studies have reported decreased *NCOA4* expression in both prostate intraepithelial neoplasia and malignant prostate when compared to benign prostate [38,39], which is consistent with our result of analysis on TCGA dataset (Figure 6b). However, another study showed similar expression levels of *NCOA4* in both normal and prostate cancer [40]. In contrast, another study demonstrated higher *NCOA4* expression in prostate cancer tissues than benign tissue [41]. *NCOA4* affects ligand-binding specificity of the AR and interacts with PSA (prostate-specific antigen) and AR, possibly forming a tripartite complex [37]. *NCOA4* increases the sensitivity and maximum stimulation of DHT-inducing PSA promoter activity [42]. Clearly, the role of *NCOA4* in prostate cancer needs further exploration.

In summary, we applied SNPs-seq to test 903 SNPs selected from our previous prostate tissue eQTL data for their allelic protein-binding difference. This analysis identified 348 SNPs that showed significantly different protein binding between two alleles (absolute BAB score ≥0.58) with and without androgen treatment conditions. Database-based informatic analysis showed 74 SNPs have potential regulation function with epigenomic marks. We used EMSA to validate the premising SNPs selected from SNPs-seq and identified three SNPs (rs12246440, rs7077275, and rs113082846) that are respectively associated with two genes (*CTBP2* and *NCOA4*). Because the EMSA validation was just performed using one cell line (LNCaP), further functional study is needed to characterize the mechanisms of the SNPs–gene associations in prostate cancer.

## Figures and Tables

**Table 1:** Distribution of selected SNPs on 28 regions (36 genes).

Risk Regions	Genes	SNPs with eQTL Signal	SNPs LD >0.5	SNPs Selected
chr1:205,657,824-205,857,824	*RAB7L1, NUCKS1*	155	40	40
chr2:10,610,730-10,810,730 **	*NOL10*	368	286	19
chr2:20,788,265-20,988,265	*C2orf43*	74	13	13
chr2:85,677,270-85,894,297	*GGCX*	19	19	19
chr2:238,287,228-238,543,226 **	*MLPH*	383	255	9
chr3:87,010,674-87,341,497	*CHMP2B*	73	17	17
chr4:95,414,609-95,662,877	*BMPR1B*	225	166	166
chr5:1,795,829-1,995,829 *	*IRX4*	71	12	71
chr6:41,436,427-41,636,427	*FOXP4*	54	43	43
chr6:76,395,882-76,595,882	*MYO6*	171	63	63
chr6:153,341,079-153,541,079	*RGS17*	330	76	76
chr7:97,716,327-97,916,327	*BHLHA15, LMTK2*	304	75	75
chr10:51,424,971-51,649,496	*NCOA4, MSMB*	156	135	135
chr10:126,596,872-126,796,872 *	*CTBP2*	99	25	99
chr11:2,133,574-2,333,574 *	*ASCL2*	53	53	53
chr11:102,301,661-102,501,661	*MMP7*	69	3	3
chr12:48,138,757-48,519,618	*COL2A1*	15	15	15
chr12:53,173,904-53,373,904	*KRT8, KRT18*	62	49	49
chr12:114,585,571-114,785,571	*TBX5*	3	3	3
chr14:61,022,526-61,222,526 **	*C14orf39*	445	243	2
chr17:518,965-718,965	*VPS53, FAM57A, GEMIN4*	513	60	60
chr17:35,974,979-36,201,156 *	*HNF1B*	21	18	21
chr19:38,635,613-38,835,613	*PPP1R14A*	278	17	17
chr19:41,885,587-42,085,624	*PCAT19*	50	20	20
chr20:62,262,563-62,462,563	*LIME1, ZGPAT*	105	104	104
chrX:9,714,135-9,914,135	*SHROOM2, GPR143*	62	40	40
chrX:51,110,057-51,341,672 **	*NUDT11*	1226	362	2
chrX:70,307,983-70,507,983	*GJB1*	59	40	40
	Total SNPs	5443	2252	1274

Notes: * all significant eQTL SNPs were selected in these regions. ** only SNPs with ChIP-seq (chromatin immunoprecipitation sequencing) signal were selected in these regions. LD: linkage disequilibrium.

**Table 2:** Comparison of EMSA with SNPs-seq for rs12246440.

	Oligo Amount	Bound/Unbound oligo		C/T Ratio
		C Allele	T Allele	
EMSA	0.1 pmol	0.012	0.023	0.522
	0.5 pmol	0.008	0.012	0.667
SNPs-seq (DHT)				0.400
SNPs-seq (ETH)				0.378

## Supplementary Materials

The following are available online at https://www.mdpi.com/2073-4425/10/7/547/s1, Figure S1: The correlation of log2 read count in overlapped SNPs between this study (903 SNPs) and previous SNPs-seq (374 SNPs). Figure S2: Distance of selected 348 SNPs to transcription start site (TSS) of the nearby genes. Figure S3: Functional annotations on the 348 SNPs visualized using circos plot. Figure S4: Validation of SNP rs7077275 by EMSA. Figure S5: Validation of SNP rs113082846 by EMSA. Figure S6: Association of RFS with combined expression value of CTBP2 and NCOA4 by log-rank test. Table S1: List of 903 SNPs, sequence read count, and BAB score. Table S2: Priority of SNPs selection based on BAB score. Table S3: Functional analysis of 348 significant SNPs using Regulome DB, HaploReg, SNPnexus, VEP, and MATCH datasets. Table S4: Functional annotations of three validated SNPs.
