# Supplementary material for: Single-Nucleotide Polymorphisms Sequencing Identifies Candidate Functional Variants at Prostate Cancer Risk Loci
Source: Genes (Basel). 2019 Jul 18;10(7):547. doi: 10.3390/genes10070547 (PMC6678189; doi:10.3390/genes10070547)
Supplement: Supplementary file 1 [file genes-10-00547-s001.zip › Supplementary_files/Supplementary-Figures-and-Tables.pdf]

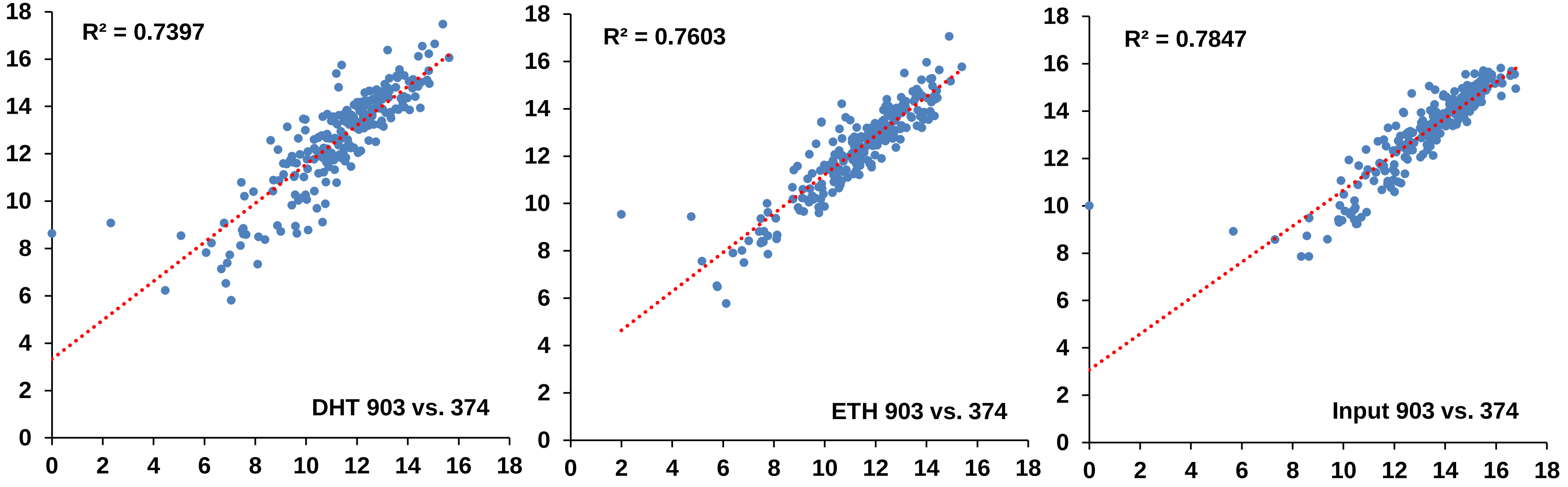

Figure S1. The correlation of log2 read count in overlapped SNPs between this study (903 SNPs) and previous SNPs-seq (374 SNPs). 116 SNPs are shared between the two studies.

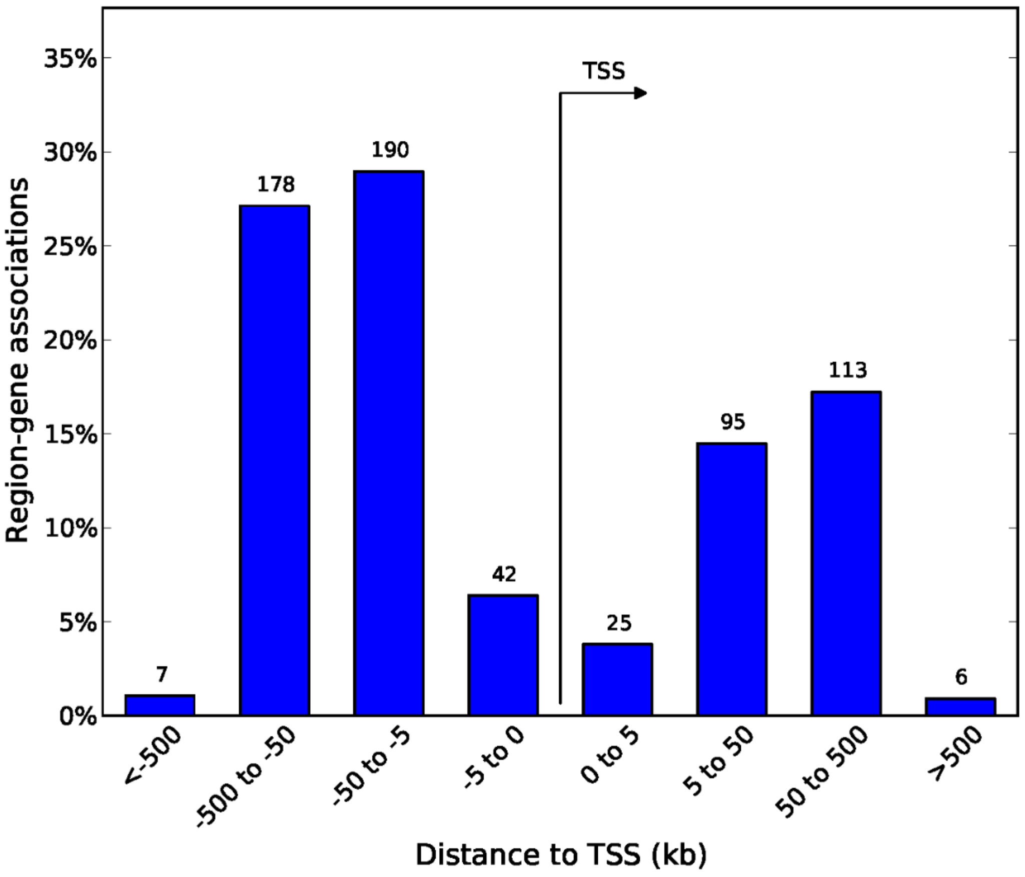

Figure S2. Distance of selected 348 SNPs to transcription start site (TSS) of the nearby genes.

Figure S3. Circos Plot showing functional annotations on the 348 SNPs. Left semicircle: from outer to inner ring are chromosomes and related SNPs, genes, and Regulome DB score ≤4. Right semicircle: Histone and transcription factor marks from HaploReg, SNPnexus, and VEP.

See Tif file

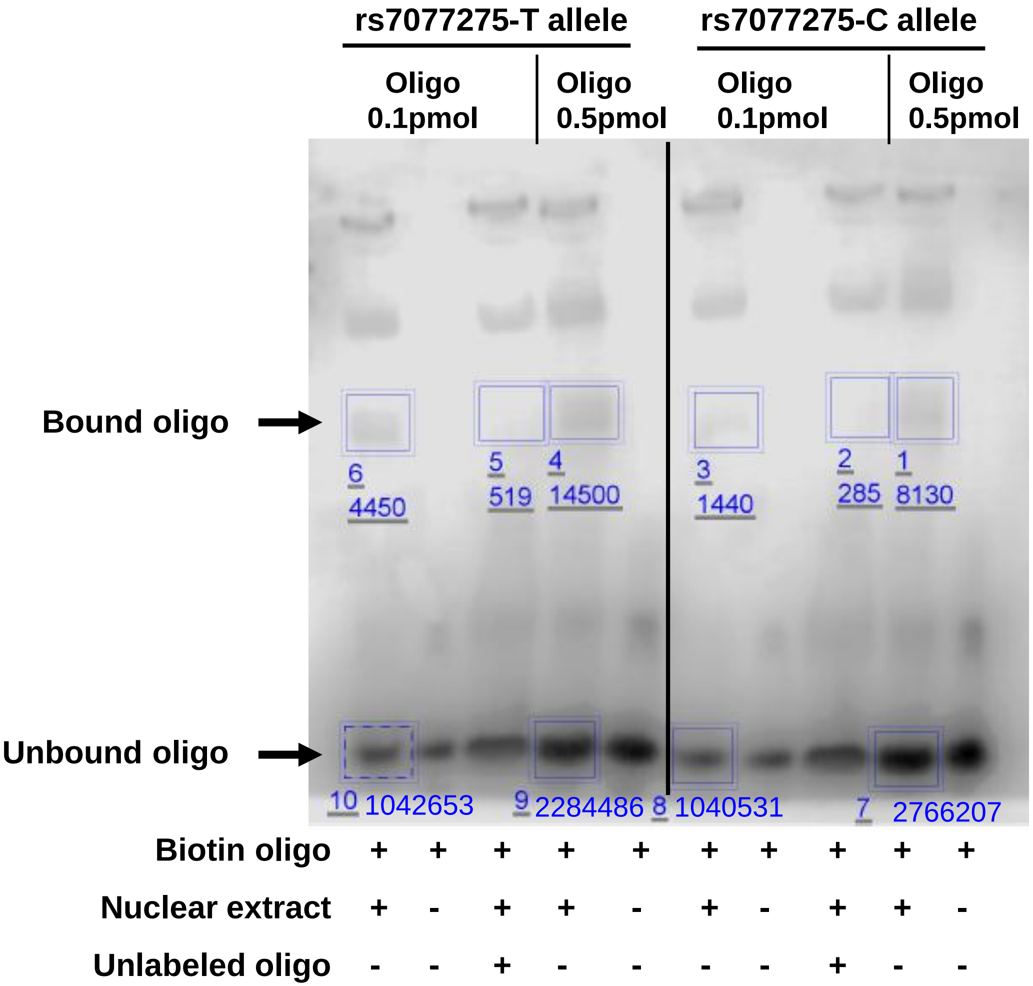

Bound/Unbound oligo	Bound/Unbound oligo	C/T Gel shift 0.1pmol	C/T Gel shift 0.5pmol
C 0.1pmol	C 0.5pmol	0.326	0.460
0.0014	0.0029		
T 0.1pmol	T 0.5pmol	**C/T SNPs-seq DHT**	**C/T SNPs-seq ETH**
0.0043	0.0063	0.496	0.573

Figure S4. Validation of SNP rs7077275 by EMSA. Compared to T allele, C allele shows weaker protein binding in both 0.1 pmol or 0.5 pmol oligo. The binding signal is reduced by addition of unlabeled oligos. The ratio of C to T allele from EMSA is consistent with ratio from SNPs-seq.

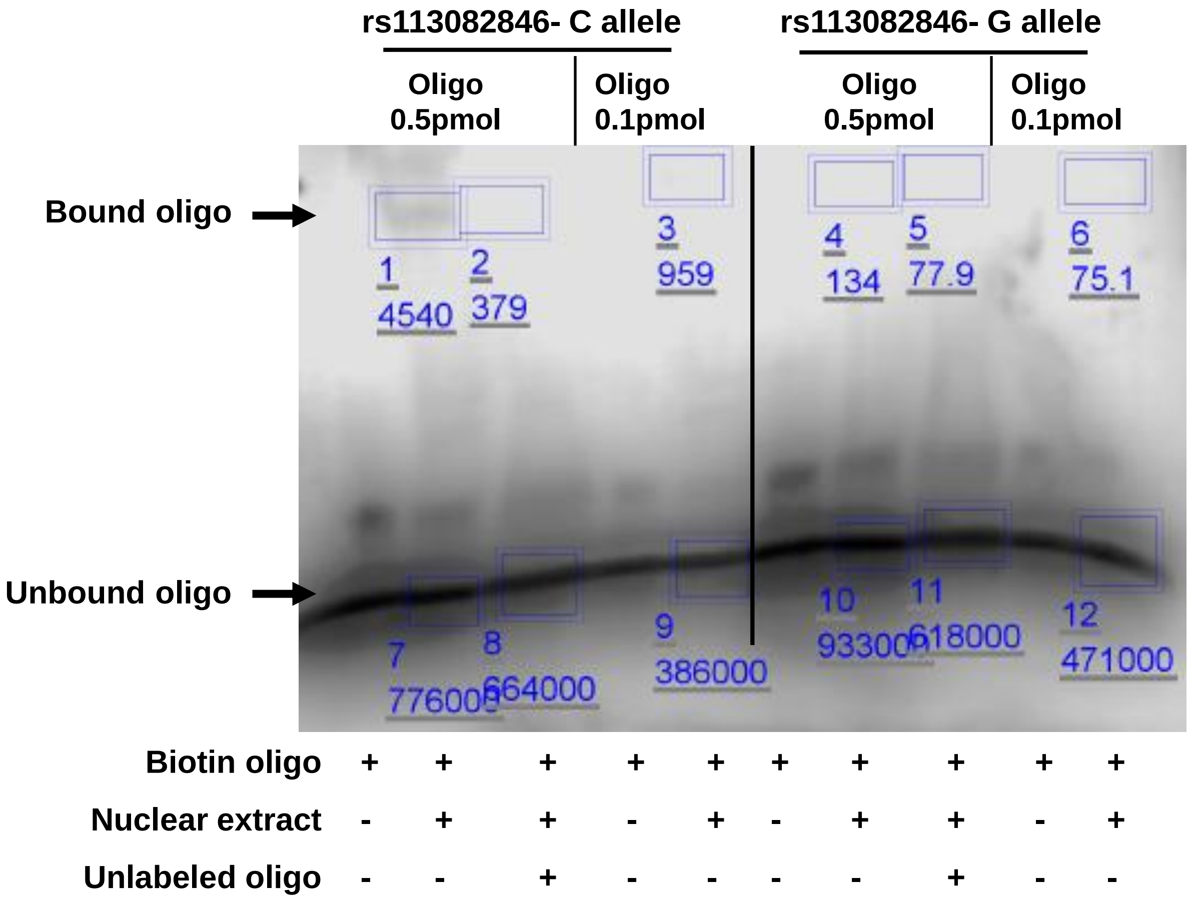

Bound/Unbound oligo	Bound/Unbound oligo	C/G gel shift 0.5pmol	C/G gel shift 0.1pmol
C 0.5pmol	C 0.1pmol	42.143	15.625
0.0059	0.0025		
G 0.5pmol	G 0.1pmol	**C/G SNPs-seq DHT**	**C/G SNPs-seq ETH**
0.00014	0.00016	7.752	8.786

Figure S5. Validation of SNP rs113082846 by EMSA. Compared to G allele, C allele shows stronger protein binding in both 0.5 pmol or 0.1 pmol oligo. The binding signal is reduced by addition of unlabeled oligos. The ratio of C to G allele from EMSA is consistent with ratio from SNPs-seq.

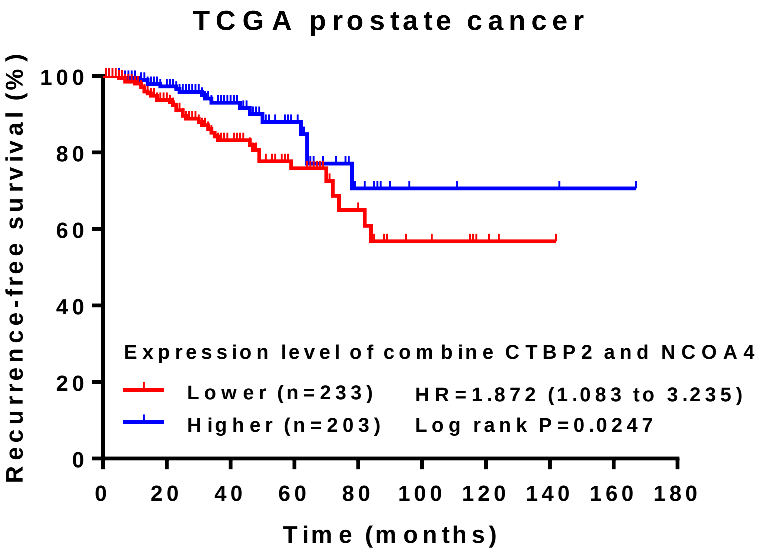

Figure S6. Association of RFS with combined expression value of CTBP2 and NCOA4 by Log-rank test.

Table S1.
List of 903 SNPs, sequence read count, and BAB score.

See Excel file

Table S2. Priority of SNPs selection based on BAB score.

Absolute BAB score	Ratio fold	DHT		Overlap	ETH	
		total	specific		total	specific
≥2.32	5	25	5	20	33	13
≥2	4	28	12	16	25	9
≥1.58	3	51	19	32	58	26
≥1	2	135	42	93	132	39
≥0.58	1.5	141	50	91	155	64
Total		380	32	348	403	55

Table S3.
Functional analysis of 348 significant SNPs using Regulome DB, HaploReg, SNPnexus, VEP and MATCH datasets.

See Excel file

Table S4. Functional annotations of 3 validated SNPs.

SNP	Gene	Regulome DB score	Histone marks	TF marks	Motif changes
rs12246440	CTBP2	4	H4K20me1, H3K79me2, H3K4me1, H3K36me3, H3K27me3, H3K14ac, H3K27ac, H2AK5ac, H4K8ac	CTCF, USF1, RAD21, JUND, YY1, GABP, SMC3, ZNF143, ATF3, BCL3, CJUN, CMYC, CTCFL, MAX	AP-1, AP-2, BAF155, BATF, BCL, Bach1, Bach2, GATA, GR, HMGN3, KAP1, Maf, Mef2, Myc, PRDM1, RXRA, STAT, TCF4, p300
rs7077275	CTBP2	2b	H3K36me3, H4K20me1, H3K27me3, H3K4me1, H3K27ac, H3K79me2	AR	ATF3, CTCF, RXRA, Rad21, SIX5, SMC3, TBX5, Tgif1
rs113082846	NCOA4	3a	H3K4me1, H3K27ac, H3K9ac, H3K27me3, H3K4me3	POU2F2, RUNX3	BCL, ELF1, Ets, PLAG1, RREB1, TATA
