# Supplementary figures and images for: Single-Nucleotide Polymorphisms Sequencing Identifies Candidate Functional Variants at Prostate Cancer Risk Loci
Source: Genes (Basel). 2019 Jul 18;10(7):547. doi: 10.3390/genes10070547 (PMC6678189; doi:10.3390/genes10070547)

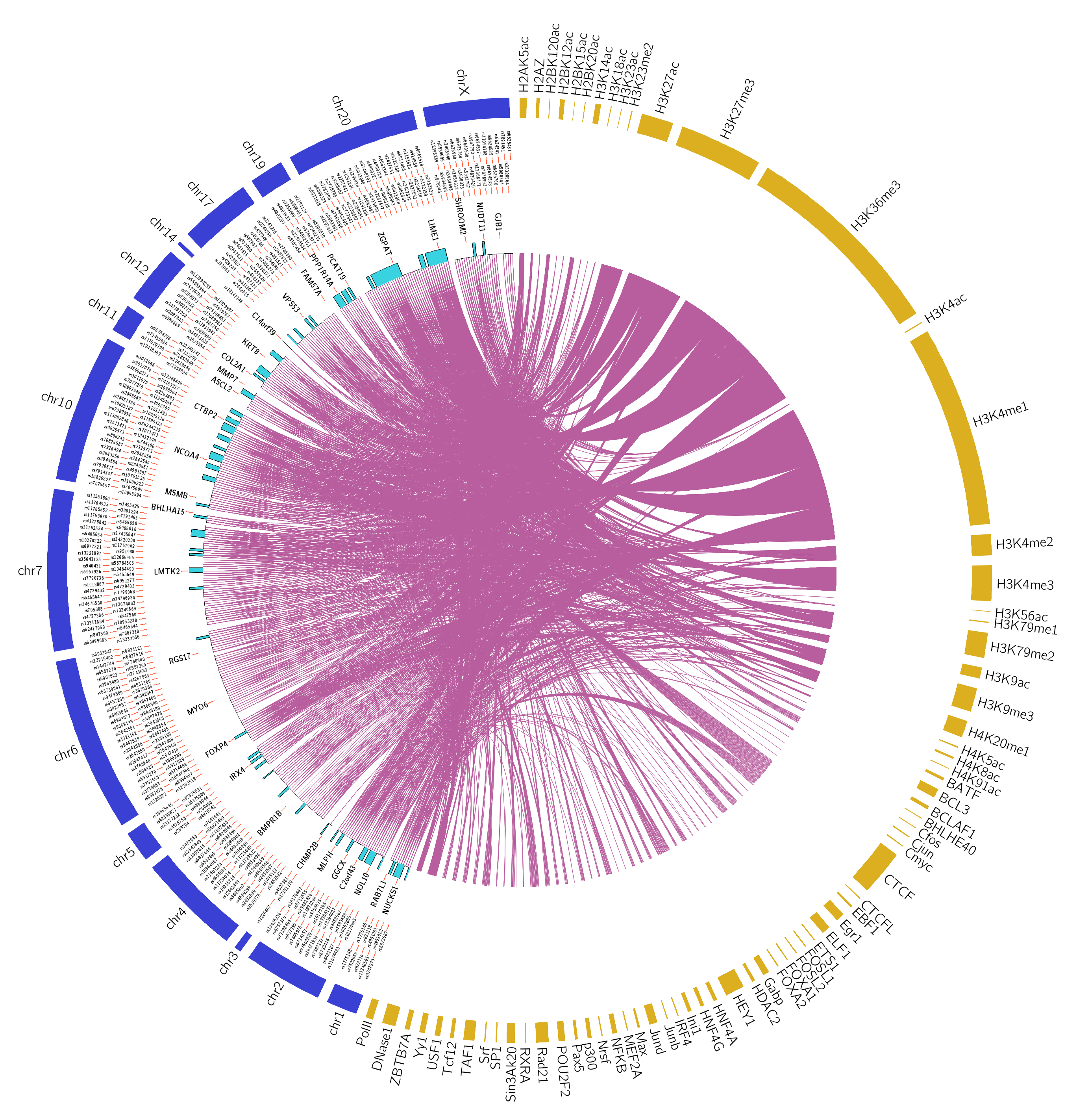

Supplement: Supplementary file 1 [file genes-10-00547-s001.zip › Supplementary_files/Figure_S3.tif]
